# Long-Term Survival and Maturation of Transplanted Cerebral Organoids Derived from *Human* Induced Pluripotent Stem Cells

**DOI:** 10.3390/cells15161466

**Published:** 2026-08-15

**Authors:** Xiaoli Ji, Zhongmeng Xiong, Wanxing Li

**Affiliations:** 1 Departments of Pneumonology, Children’s Hospital of Fudan University, Shanghai 201102, China; 15502179101@163.com; 2 Key Laboratory of Birth Defects, Children’s Hospital of Fudan University, Shanghai 201102, China; 23111240027@m.fudan.edu.cn

**Keywords:** cerebral organoids, transplantation, survival, maturation, synaptic formation, vascularization

## Abstract

**Highlights:**

**What are the main findings?**

- hiPSC-derived cerebral organoids survived and matured into cortical neuronal subtypes, GABAergic neurons, and glial cells at 12 months post-transplantation.
- Grafted cells formed synapses and achieved vascularization within the host brain.

**What are the implications of the main findings?**

- This study provides evidence of the long-term survival, multilineage differentiation, and maturation of hiPSC-derived cerebral organoid grafts.
- Synaptic formation and vascularization between grafts and hosts provide the foundation for the clinical application of brain organoids as cell therapy resources.

**Abstract:**

*Human* cerebral organoids have emerged as a promising new therapeutic strategy for cell transplantation after brain injury. Researchers have explored the short-term survival and maturation of human cerebral organoids derived from *human* embryonic stem cells (hESCs) in animal models. However, the long-term survival and maturation of cerebral organoids derived from *human* induced pluripotent stem cells (hiPSCs) have not been studied in depth. In this study, we generated cerebral organoids from hiPSCs in a feeder-free culture system. Then, the cerebral organoids were digested into small clusters and transplanted into the frontal cerebral cortex of postnatal day 0 (P0) SCID mice. The long-term survival and maturation of the grafted cerebral organoids were evaluated at 12 months post-transplantation. Our results indicate that grafted cerebral organoids survive well and maintain their forebrain identity in vivo over a long period. The transplanted cerebral organoids showed reduced proliferative capacity, indicating a low risk of tumor formation. The majority of transplanted cells differentiated into cortical neuronal subtypes in different cortical layers in anatomical lamination at 12 months post-transplantation. In addition, a small population of grafted cerebral organoids matured into GABAergic neurons and gliocytes, including astrocytes, microglia and oligodendrocytes. Furthermore, the grafts formed synapses with the host cells and achieved vascularization in the host brain. Our study demonstrates the long-term survival and maturation of cerebral organoids derived from hiPSCs in vivo and provides evidence for the feasibility of cerebral organoids derived from hiPSCs as a potential cell transplantation therapy.

## 1. Introduction

Neurological disorders such as stroke and hypoxic–ischemic encephalopathy are high-incidence health problems worldwide [1,2]. Massive brain tissue infarction, neuronal loss and gliosis occur as a result of these disorders [3]. With the advent of stem cell technology, various types of stem cells, including fetal brain tissues, neural stem cells (NSCs) and mesenchymal stem cells (MSCs), have been used for the treatment of brain injury [4,5]. Brain injury, especially cortical injury, usually involves damage to multiple cell types and even different brain regions. However, traditional monolayer (2D) cultures of stem cells usually produce relatively uniform cell populations [6,7,8]. Brain organoids differentiated from *human* embryonic stem cells (hESCs) or *human* induced pluripotent stem cells (hiPSCs) via 3D culture resemble the developing brain in structure and function [9,10,11]. The generated brain organoids exhibit well-developed cytoarchitecture, containing neural stem cells and organized neuronal subtypes in all six cortical layers [12]. Therefore, brain organoids have become a potential tool for modeling *human* brain development, cell transplantation, disease modeling, and drug screening.

The transplantation of cerebral organoids differentiated from hESCs into the *mouse* cortex was evaluated in terms of graft survival, neuronal differentiation, axonal extension, and damage repair [13,14]. The transplanted cerebral organoids showed positive expression of deep-layer markers (TBR1 and CTIP2) and the late-born upper-layer neuronal marker (SATB2) at 7 days post-transplantation. Cells from the grafted cerebral organoids survived and showed extensive migration and vascularization into the host brain at 28 days post-transplantation [15]. Another study reported that the grafted cerebral organoids expressed *human* presynaptic marker synaptophysin at 3 months post-transplantation, indicating their synaptic formation. Moreover, the engrafted organoids showed functional maturation and formed bidirectional synaptic connections with the host mouse brain at 5 months post-transplantation [16]. Studies have indicated that the maturation time of cortical neurons ranges from several weeks in *mice* to several months in *macaques* to years in *humans* [17]. A remarkable feature of *human* cortical neurons is their prolonged development of dendritic morphogenesis, synaptogenesis and synaptic pruning over months to several years. Therefore, it is necessary to explore the long-term survival and maturation of grafted cerebral organoids in vivo.

The successful generation of brain organoids from hESCs or hiPSCs raises the possibility of their application in stem cell transplantation. hiPSCs have similar self-renewal and differentiation potential to hESCs [18]. For clinical translation, hiPSCs have more advantages than hESCs in terms of immunological rejection and ethical approval. In addition, to maintain the cytoarchitectural integrity of the transplants, most studies have chosen to inject the whole organoids directly into the cavity of the mouse brain [15,19]. However, cell-dense transplants containing multiple lumens and neural tubes may have limited access to the host tissue. In addition, immature proliferating neural stem cells are still abundant in organoids and usually cause graft overgrowth after being transplanted in vivo. These oversized grafts not only compress the surrounding healthy brain tissue but are also prone to forming tumors even a long time after transplantation [20]. Therefore, alternative approaches for the transplantation of brain organoids need to be explored further. It is likely that the transplantation of brain organoids in the form of small clusters may mitigate these safety concerns and make it easier to inject them into specific brain regions.

In this study, we generated cerebral organoids derived from hiPSCs in a feeder-free culture system. The cultured cerebral organoids were dissociated and transplanted in the form of small clusters into the frontal cortex of SCID *mice* (P0 SCID *pups*). The survival and maturation of grafts were evaluated at 12 months post-transplantation. Our results demonstrated that the grafted small organoids survived well and had low proliferative capacity over a long period. The majority of the transplanted cells matured into cortical neuronal subtypes in different cortical layers, and a small population of grafts matured into GABAergic neurons and gliocytes. Moreover, we found that the grafted organoids formed synapses with the host cells and achieved vascularization within the host brain. Our study demonstrates the long-term survival and maturation of cerebral organoids derived from hiPSCs and provides pre-clinical evidence for cerebral organoids as a potential stem cell therapy for neurological disorders.

## 2. Materials and Methods

### 2.1. hiPSC Culture and Cerebral Organoid Differentiation

The FDCHi001-A hiPSC line was used in this study [21]. Briefly, breast milk cells (BMCs) were isolated from fresh breast milk donated by breastfeeding mothers. hiPSCs were generated from BMECs with Yamanaka factors (OCT4, SOX2, c-MYC, KLF4) using an episomal system. The pluripotency of breast milk-derived hiPSCs (BM-hiPSCs) was confirmed by the expression of pluripotent markers OCT4, NANOG, SOX2 and SSEA4 (Figure S1).

hiPSCs were cultured in feeder-free conditions on six-well plates using E8 medium (Life technology, Carlsbad, CA, USA), which was replaced every other day. Cerebral organoids were generated from hiPSCs following previously published methods with minor modifications [12,22]. Briefly, hiPSC colonies were dissociated into small clumps with dispase for 2 min and then re-aggregated in neural induction medium composed of DMEM/F12 (Life technology, Carlsbad, CA, USA), 1x Glutamax (Life technology, Carlsbad, CA, USA), 1x N2 (Life technology, Carlsbad, CA, USA), 1x NEAA (Life technology, Carlsbad, CA, USA), 2 μM DMH1 (Tocris Bioscience, Bristol, UK) and 2 μM SB431542 (Segment.io, Inc., San Francisco, CA, USA). The aggregates were cultured in T25 flasks (Corning Incorporated, NY, USA) for 3 days to allow for embryoid body (EB) formation. On day 4, half of the medium was replaced with cortical differentiation medium consisting of DMEM/F12, 1x Glutamax, 1x N2, 1x NEAA, 1 µM CHIR (Tocris Bioscience, Bristol, UK), and 1 µM SB431542. On days 5 to 7, the culture medium was completely replaced by cortical differentiation medium. On day 7, EBs were embedded in Matrigel (Corning Incorporated, NY, USA) and cultured in cortical differentiation medium for another 7 days in ultra-low attachment six-well plates, during which neural buds gradually extended to form organoid structures. On day 14, the embedded organoids were dissociated from Matrigel mechanically. Approximately 20 organoids were transferred to each well of ultra-low attachment six-well plates (Corning Incorporated, NY, USA) with differentiation medium, consisting of DMEM/F12, 1x N2, 1x B27 (Life technology, Carlsbad, CA, USA), 1x NEAA, 1x Glutamax, 1x 2-mercaptoethanol (Sigma-Aldrich Corporation, St. Louis, MO, USA), and 2.5 μg/mL Insulin (PeproTech, Inc., Cranbury, NJ, USA). From day 14, the six-well plates with organoids were placed on an orbital shaker to promote culture maturation. From day 35 to day 70, Matrigel was supplemented into the differentiation medium at a 1:100 ratio. From day 70, the medium was switched to maturation medium consisting of neurobasal (Life technology, Carlsbad, CA, USA), 1x N2, 1x B27, 1x Glutamax, 1x NEAA, 0.5 mM cAMP (Sigma-Aldrich Corporation, St. Louis, MO, USA), 0.2 mM AA (Sigma-Aldrich Corporation, St. Louis, MO, USA), and 20 ng/mL BDNF (PeproTech, Inc., Cranbury, NJ, USA), and 20 ng/mL GDNF (PeproTech, Inc., Cranbury, NJ, USA) was added. The media were changed every 2–4 days throughout the culture period.

### 2.2. Ethics Statement and Animals

This study was conducted in accordance with the National Institute of Health’s Guide for the Care and Use of Laboratory Animals. All animal experiments were approved by the Research Ethics Board of the Children’s Hospital of Fudan University (Agreement number: 2023-EKYY-37JZS). The work has been reported in line with the ARRIVE guideline 2.0. Severe combined immunodeficiency (SCID) *mice* were purchased from Beijing Vital River Laboratory Animal Technology Co., Ltd. (Beijing, China), housed in a climate-controlled room under a 12 h light/12 h dark cycle and had free access to food and water throughout the study. Following free mating, the time of delivery was estimated based on the detection of a vaginal plug. The postnatal day (P0) SCID *pups* underwent the cell transplantation procedure.

### 2.3. Cell Transplantation

On day 33, cerebral organoids were digested into single cells. Following one day of culture, these single cells reaggregated into many small clusters, which were collected for transplantation on day 35. P0 SCID *pups* (*n* = 10) received an intracranial injection of 1.5 μL of the day 35 cell clusters via a glass micropipette. Briefly, the cell clusters were suspended in artificial cerebrospinal fluid (aCSF) (Tocris Bioscience, Bristol, UK) containing 1x B27, 20 ng/mL BDNF, and 0.5 μM ROCK inhibitor (Calbiochem-Novabiochem Corp., San Diego, CA, USA), and 1.5 μL of this suspension was injected into the left cortex at the following stereotaxic coordinates: AP: +1.0 mm; ML: +1.0 mm; DV: −1.0 mm. After injection, the *pups* were returned to their dams and reared under standard conditions for subsequent analyses. All the *pups* survived well after the transplantation surgery. The final survival rate of transplanted animals reached 80% at 12 months post-transplantation. Brain sections from the eight surviving *mice* were analyzed. Among these, one *mouse* exhibited no cell survival at the graft site, and another *mouse* was excluded due to off-target injection into the lateral ventricle. Thus, six *mice* were ultimately included in the final statistical analysis.

### 2.4. Immunofluorescence and Quantification

For immunofluorescence, cultured cerebral organoids were rinsed with DPBS (Life technology, Carlsbad, CA, USA) and fixed in 4% paraformaldehyde (PFA) (Sigma-Aldrich Corporation, St. Louis, MO, USA) for 30 min or 1 h. After fixation, the cerebral organoids were embedded in optimal cutting temperature (OCT) (Sakura Finetek, Torrance, CA, USA) compound and cut into 14 μm thick sections using a freezing microtome (Leica Microsystems GmbH, Wetzlar, Germany). For immunostaining, sections were permeabilized and blocked in 10% donkey serum (Life technology, Carlsbad, CA, USA) containing 0.2% Triton X-100 (Sigma-Aldrich Corporation, St. Louis, MO, USA) for 1 h at room temperature. After blocking, sections were incubated with primary antibodies overnight at 4 °C, followed by incubation with fluorescently conjugated secondary antibodies for 1 h at room temperature (Table S1). Nuclei were stained with Hoechst 33342 (Life technology, Carlsbad, CA, USA) and slices were mounted in Fluoromount-G (Southern Biotechnology, Birmingham, AL, USA). Images were acquired using a Nikon TIE A1 plus laser scanning confocal microscope (Nikon, Tokyo, Japan).

### 2.5. RNA Isolation and Real-Time PCR

Total RNA was extracted from cultured organoids with Trizol reagent (Invitrogen Corporation, Carlsbad, CA, USA). cDNA was generated from 1 μg of total RNA using Prime Script RT Master Mix (Takara Bio Inc., Kusatsu, Shiga, Japan). Real-time PCR (RT-PCR) was performed using a SYBR Green PCR kit (Takara Bio Inc., Kusatsu, Shiga, Japan). All reactions were performed in duplicate and amplified using the Light Cycler 480 real-time PCR System (Roche, Basel, Switzerland). Finally, gene expression levels were normalized to the housekeeping gene (GAPDH) using the ΔΔCT method. The primer sequences are supplied in Table S2.

### 2.6. Tissue Preparation and Immunohistochemistry

At 12 months post-transplantation, the SCID *mice* were perfused transcardially with 0.9% saline (Life technology, Carlsbad, CA, USA) and then fixed with 4% PFA for 2 h. After dehydration in 20% sucrose (Life technology, Carlsbad, CA, USA) for 2 days, followed by 30% sucrose for another 2 days, the mouse brains were sectioned into 30 μm thick slices using a freezing microtome. For immunohistochemical staining, brain slices were incubated with blocking solution (10% donkey serum and 0.3% triton X-100 in DPBS) for 1 h, and then incubated with primary antibodies at 4 °C overnight. After washing with DPBS, the slices were incubated with the corresponding fluorophore-conjugated secondary antibodies for 1 h at room temperature. Nuclei were stained with Hoechst 33342, and slices were mounted in Fluoromount-G. Images were captured using a Nikon TIE A1 plus confocal microscope. Serial brain sections (every fifth section within the graft region) were selected for stereological quantification of transplanted cells. The transplanted cerebral organoids were identified by positive staining for human nuclei (hN). Cell counting was performed using Image J software (version 1.54p). Data are presented as the mean ± SEM.

### 2.7. Statistical Analysis

All data were analyzed descriptively; results are expressed as the mean ± SEM. No statistical comparisons were made between groups.

## 3. Result

### 3.1. Generation and Characterization of Cerebral Organoids Differentiated from hiPSCs in Feeder-Free Culture System

**Figure 1:**
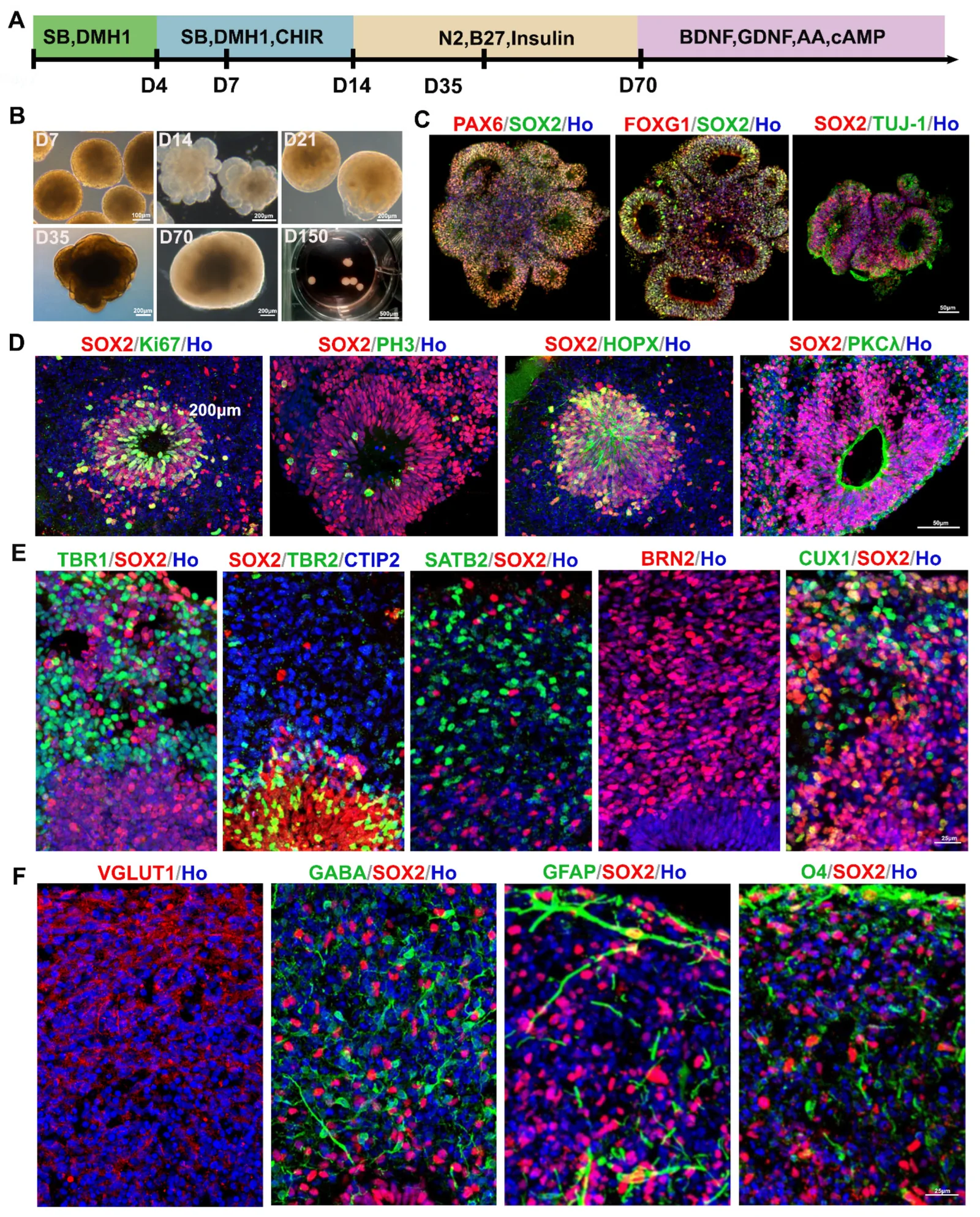
Generation and characterization of cerebral organoids derived from hiPSCs at different stages. (**A**) Schematic diagram showing the entire cerebral organoid culture procedure in vitro. (**B**) Representative bright-field images of cultured cerebral organoids at various differentiation stages. (**C**,**D**) Representative immunofluorescent images displaying the expression of PAX6, SOX2, FOXG1, Tuj-1, Ki67, PH3, HOPX and PKCλ in 2-week-old organoids in vitro. (**E**) Representative immunofluorescent images displaying the expression of TBR1 (layer VI), CTIP2 (layer V), SATB2 (layer IV), BRN2 (layer III), and CUX1 (layer II) in 10-week-old organoids. (**F**) Representative immunofluorescent images showing the expression of VGLUT1, GABA, GFAP and O4 in 15-week-old organoids.

To generate cerebral organoids from hiPSCs, we modified the differentiation protocol based on previously published methods [12,22]. hiPSCs were cultured under feeder-free conditions. Embryoid bodies (EBs) were patterned toward a specific brain region using neural induction medium containing dual SMAD inhibitors (SB431542 and DMH1). From days 7 to 14, the EBs were embedded in Matrigel, and the neuroepithelial buds were extended to form brain organoids (Figure 1A,B). Meanwhile, neural tube-like structures expressing neural progenitor markers (PAX6, SOX2, Tuj-1) were formed during this stage. The hallmarks of cerebral organoid formation include the establishment of forebrain identity, well-developed neuroepithelial ventricular zones, and six organized cortical neuronal layers [23]. In our study, positive staining for the forebrain marker FOXG1 confirmed the successful generation of forebrain cerebral organoids (Figure 1C). From day 14, the cerebral organoids cultured in ultra-low attachment six-well plates were placed on an orbital shaker. A stratified neuroepithelium-like architecture was gradually formed, with the expression of proliferation markers (Ki67 and PH3) and the adherents junction marker PKCλ. In addition, we observed the presence of HOPX-positive cells near the ventricular surface, indicating the generation of outer radial glia cells (oRGCs) in the cultured cerebral organoids (Figure 1D). At day 70, we analyzed the expression of markers for different cortical neuronal subtypes using immunofluorescent staining. Neurons expressing deep-layer (TBR1, CTIP2) and upper-layer (SATB2, BRN2, CUX1) cortical markers suggested that the cultured cerebral organoids resembled the cytoarchitecture of the developing human brain (Figure 1E). The expression of VGLUT1 and GABA indicated the successful generation of cortical excitatory glutamatergic neurons and inhibitory GABAergic interneurons from the cerebral organoids. It has been reported that, during mammalian cerebral cortex development, neurons are generated first, followed by oligodendrocytes and astrocytes [24]. In our study, positive staining for O4 and GFAP indicated the presence of oligodendrocytes and astrocytes at day 105 (Figure 1F).

For the in vitro organoid studies, we also performed quantitative RT-PCR analysis on organoids at various culture stages. The results of RT-PCR revealed that neural progenitor cell-associated genes (*PAX6*, *TBR2*) were mainly expressed in the early stage of the organoids. Meanwhile, the genes related to mature neurons (*TBR1*, *CTIP2*, *SATB2*) and the glial cell marker (*GFAP*) gradually increased with prolonged in vitro culture (Figure S2).

Taken together, our data demonstrate the successful generation of cerebral organoids with stratified cortical architecture from hiPSCs in a feeder-free culture system in vitro.

### 3.2. Grafted Cerebral Organoids Maintain Forebrain Identity and Exhibit Reduced Proliferative Capacity at 12 Months Post-Transplantation

To explore the long-term survival and maturation of hiPSC-derived cerebral organoids after transplantation in vivo, we dissociated the cerebral organoids into single cells on day 33 and transplanted the aggregated small clusters into the forebrain cortex of postnatal day 0 (P0) SCID *mice* on day 35. Before cell transplantation, we assessed the initial state of in vitro organoids at day 35 by immunofluorescent staining. Our results showed that the organoids at this stage still highly expressed the neural progenitor markers SOX2, PAX6, and TBR2, as well as the proliferation markers Ki67 and PH3 (Figure S3A). In addition, the neuronal markers MAP2 and CTIP2 began to be expressed at this stage (Figure S3B,C). These results indicated that the organoids were still in an immature state on day 35.

**Figure 2:**
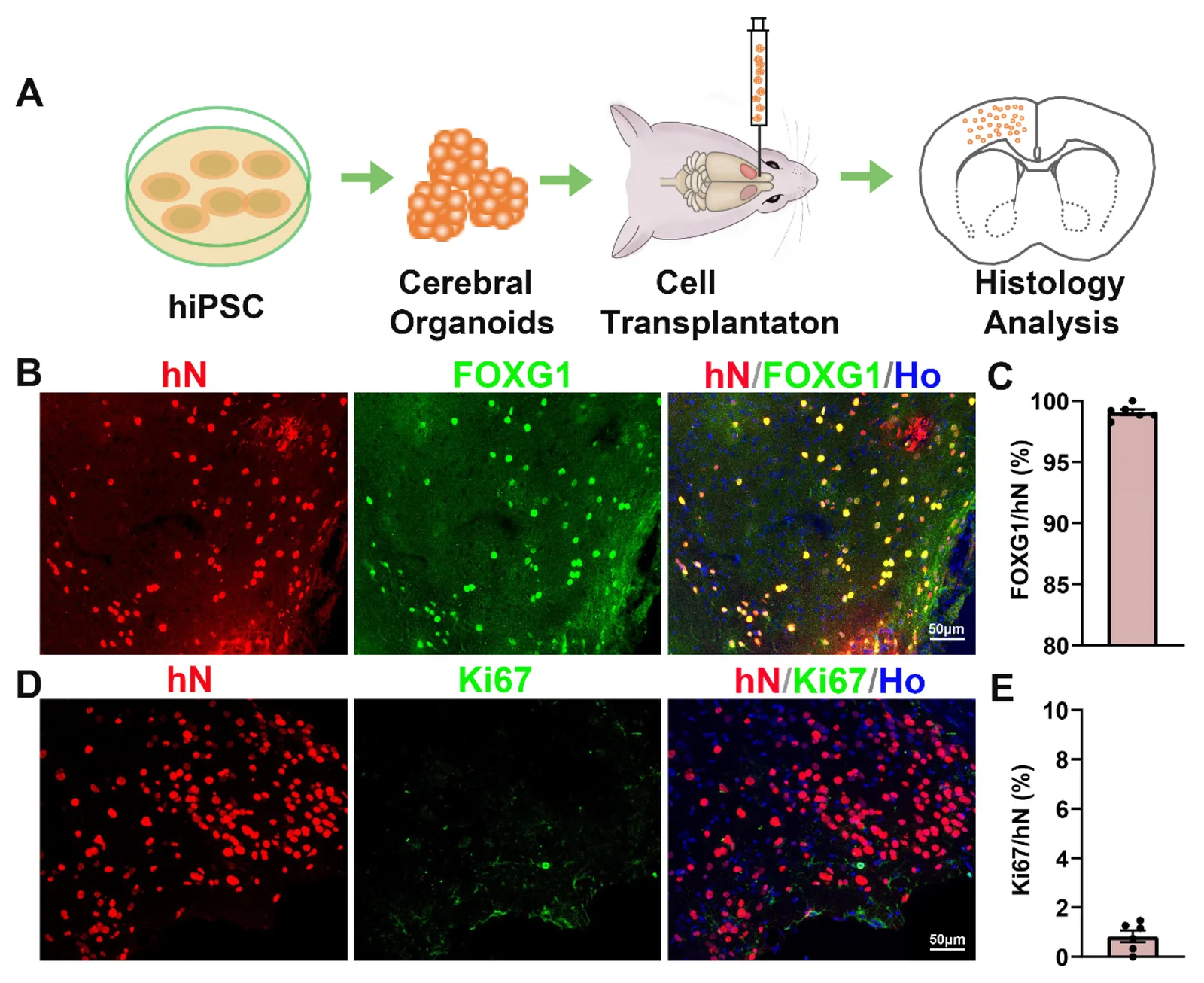
Engrafted cerebral organoids maintained forebrain identity and showed less proliferation ability at 12 months post-transplantation. (**A**) Schematic diagram showing the differentiation, cell transplantation, and histology analysis of grafted cerebral organoids in SCID mice. (**B**,**C**) Immunofluorescent staining and quantification for FOXG1 showed the forebrain identity of grafted cerebral organoids. (**D**,**E**) Immunofluorescent staining and quantification for Ki67 showed low proliferation ability at 12 months post-transplantation. Data are presented as mean ± SEM.

The proliferative ability and tumorigenicity of transplanted cells are concerns that have always affected the success of cell transplantation. We evaluated the proliferation of grafted cells by staining for Ki67. At 12 months post-transplantation, the *mice* were euthanized for immunohistochemical analysis (Figure 2A). The grafted cerebral organoids were identified by positive staining for human nuclei (hN). Data analysis revealed that 99.07% ± 0.58% of transplanted cells were positive for FOXG1, indicating the maintenance of forebrain identity after long-term maturation in vivo (Figure 2B,C). Only 0.83% ± 0.58% of grafted cells were positive for Ki67, indicating a low proliferative capacity and low tumorigenic potential (Figure 2D,E).

### 3.3. Grafted Cerebral Organoids Differentiate into Cortical Neuronal Subtypes at 12 Months Post-Transplantation

**Figure 3:**
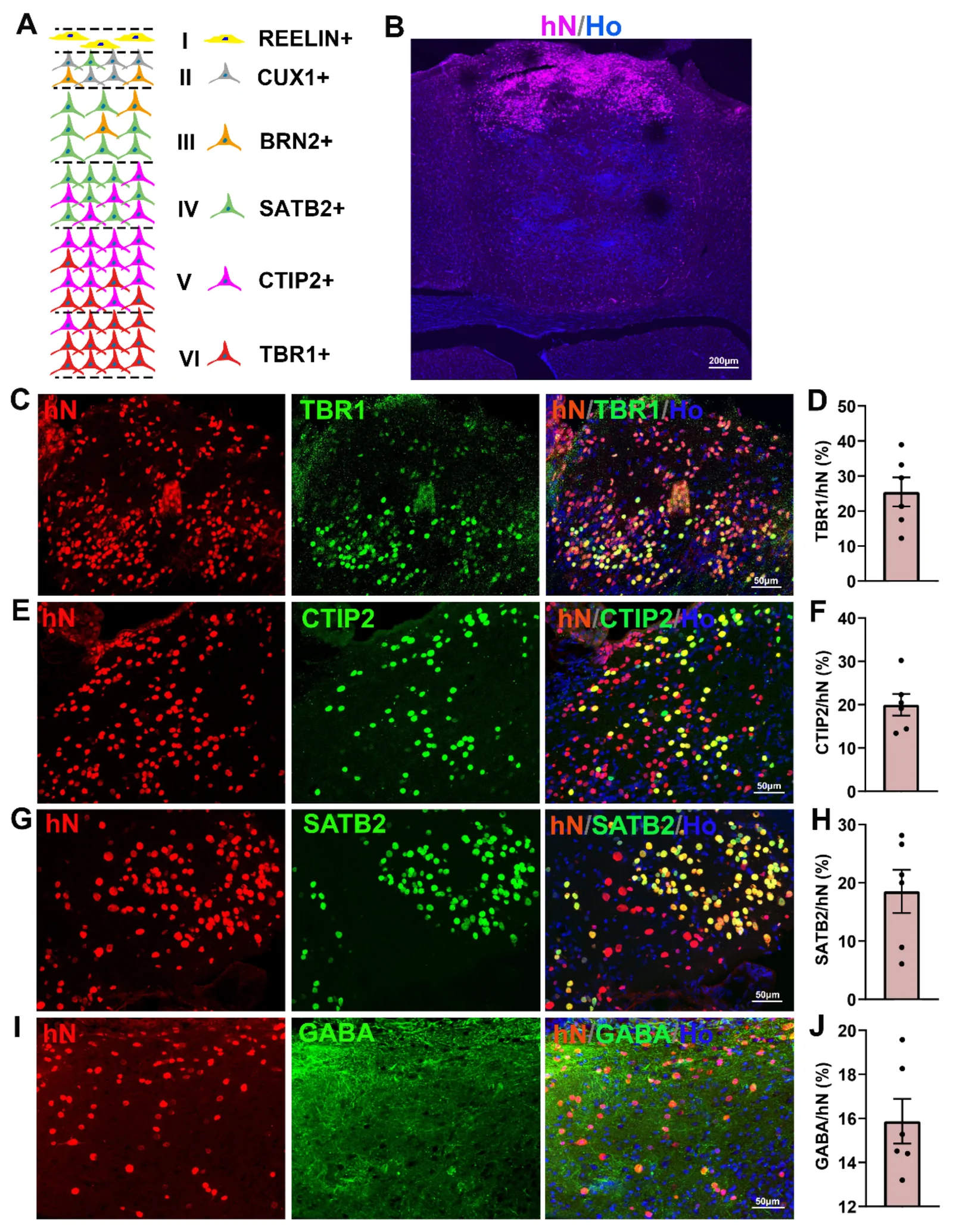
Engrafted cerebral organoids differentiated into cortical neuron subtypes at 12 months post-transplantation. (**A**) Schematic diagram showing the expression of cortical neuron markers in the mature mammalian neocortex. (**B**) Representative immunofluorescent images displaying the hN-positive graft area. (**C**,**E**,**G**,**I**) Immunofluorescent images showing the TBR1-, CTIP2-, SATB2-, and GABA-positive cells in the grafts at 12 months post-transplantation. (**D**,**F**,**H**,**J**) Quantification of cellular composition presented in (**C**,**E**,**G**,**I**). Data are presented as mean ± SEM.

Next, we analyzed the expression of markers for cortical neuronal subtypes in different layers 12 months after transplantation (Figure 3A). The low-magnification image shows that the transplanted cells were distributed within the cortex of the host brain at 12 months post-transplantation. The area of the graft was identified according to the presence of human nuclei (hN)-positive cells (Figure 3B). In the mammalian cerebral cortex, deep-layer cortical neurons expressing the markers TBR1 and CTIP2 are located in cortical layers V–VI. TBR1 is a transcriptional regulator that is involved in the developmental process of early-born neurons and is predominantly confined to cortical neurons in layer VI [25]. CTIP2 is expressed in layer V and plays an essential role in the specification of subcortical projection neuron fates [26]. In our study, we observed that 25.46% ± 10.13% of transplanted cells were TBR1-positive, and 19.98% ± 6.09% were CTIP2-positive. Upper-layer cortical neurons expressing the markers SATB2, BRN2, CUX1 and REELIN are located in cortical layers I–IV. In our study, we analyzed the expression of SATB2, which is expressed in a subset of postmitotic neurons in layers II–IV. We found that 18.53% ± 9.11% of the transplanted cells were SATB2-positive. Through immunofluorescent staining for TBR1, CTIP2 and SATB2, we found that the TBR1- and CTIP2-positive deep-layer neurons were mainly distributed in the deep cortical layers (Figure S4A,B), while the SATB2-positive neurons were mainly distributed in the upper cortical layers (Figure S4C). We speculate that this anatomical lamination is mainly attributable to the migratory characteristics inherent in neuronal maturation.

In addition to glutamatergic neurons, GABAergic neurons are distributed throughout all six cortical layers and form the major inhibitory system in the mammalian cortex. In our in vitro culture system, we found that GABA-positive cells were distributed in different layers within the cerebral organoids. After transplantation in vivo, approximately 15.87% ± 2.49% of transplanted cells were GABA-positive.

### 3.4. Grafted Cerebral Organoids Differentiate into Glial Cells at 12 Months Post-Transplantation

**Figure 4:**
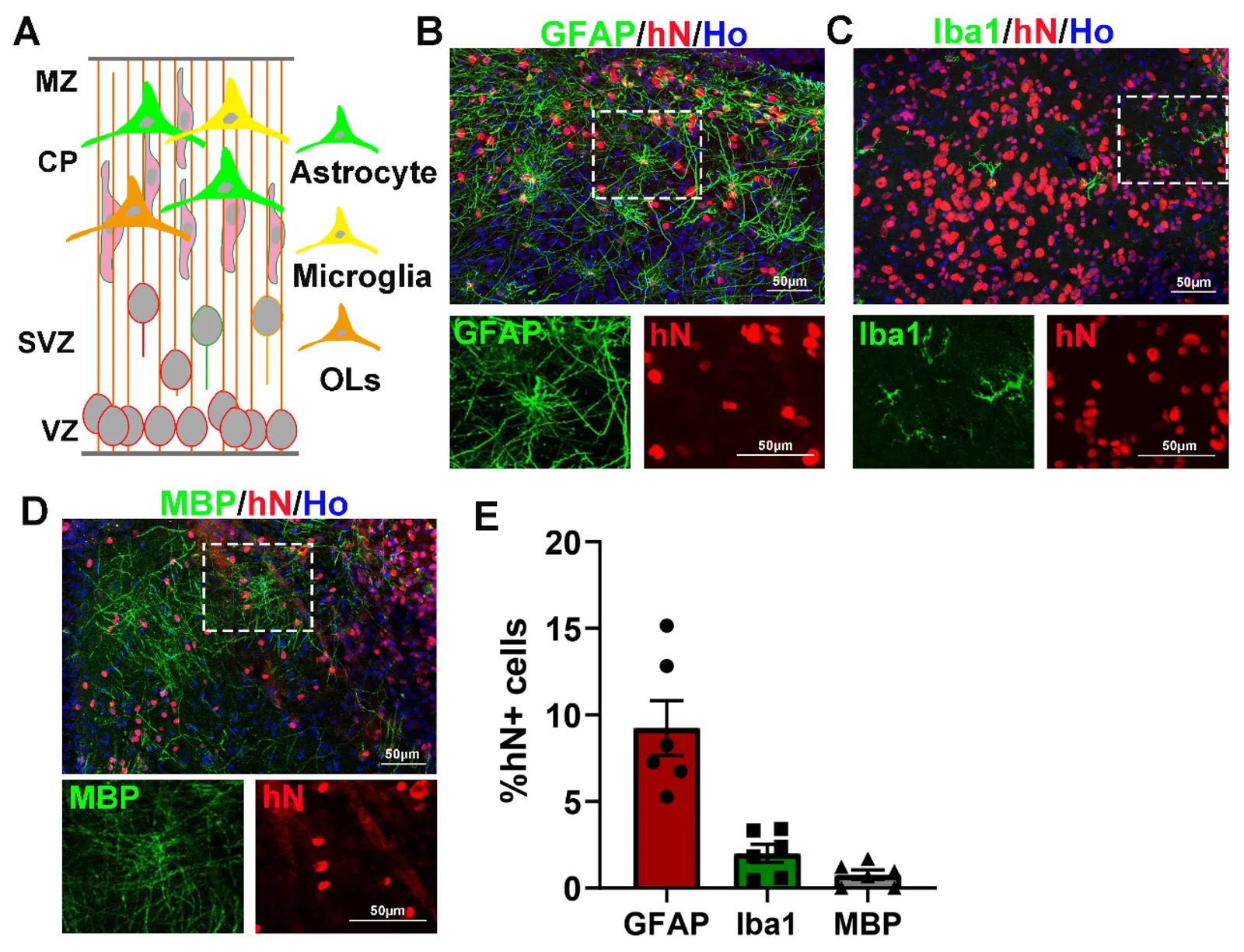
Engrafted cerebral organoids differentiated into gliocytes at 12 months post-transplantation. (**A**) Schematic diagram showing the expression of gliocyte markers in the mature mammalian neocortex. (**B**–**D**) Representative immunofluorescent images showing GFAP-, IBA-1-, and MBP-positive cells in the grafts at 12 months post-transplantation. The boxed area is magnified below. (**E**) Quantification of cellular composition presented in (**B**–**D**). Data are presented as mean ± SEM.

Glial cells play an important role in neurotrophic support, information processing, and integration into neural networks with neurons (Figure 4A). In our study, we observed that a subset of transplanted cells were glial cells that were closely associated with the surrounding neurons. Among them, 9.25% ± 3.87% expressed the astrocyte marker GFAP (Figure 4B). These GFAP-positive cells exhibited numerous processes, forming well-delineated bushy morphologies. In addition, 1.99% ± 1.29% of transplanted cells, which exhibited ramified processes and small somata, expressed the microglial marker Iba1. Only a small population of transplanted cells expressed the mature oligodendrocyte marker MBP (0.76% ± 0.68%).

### 3.5. Synapse and Vascular Formation Between Grafts and Host Cells in Mouse Brains

**Figure 5:**
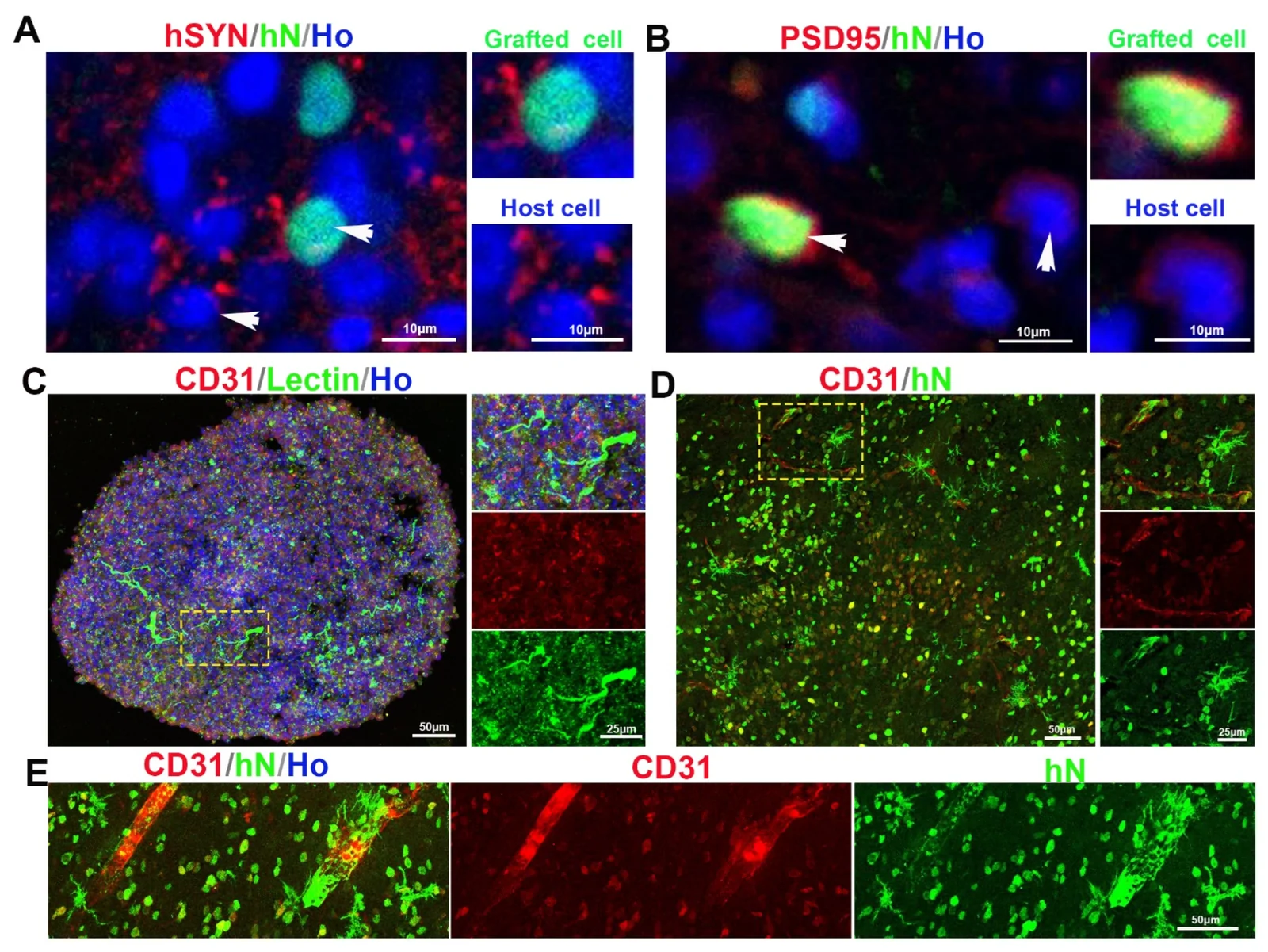
Synapse and vascular formation between grafts and hosts in mouse brains. (**A**) Representative images show presynaptic human synaptophysin (hSYN) costained with grafted cells and host cells in the cortex. White arrow indicates the magnified area shown on the right. (**B**) Representative images show postsynaptic marker PSD95 costained with grafted cells and host cells in the cortex. (**C**) Immunofluorescent staining for the endothelial markers CD31 and lectin in 10-week-old organoids in vitro. Boxed areas are magnified in the right three columns. (**D**,**E**) Immunofluorescent staining for CD31 and hN in grafts at 12 months post-transplantation. Boxed areas are magnified in the right three columns.

To further investigate whether the grafted neurons could anatomically integrate into the host neural circuits, we performed double immunostaining for the *human*-specific presynaptic marker synaptophysin (hSYN) and the postsynaptic marker PSD95 on both grafted and host cells. As shown in Figure 5A,B, hSYN and PSD95 puncta were detected in the host brain, suggesting the establishment of structural synaptic connectivity between the grafted and host cells.

Next, we further evaluated the vascularization of cerebral organoids derived from hiPSCs both in vivo and in vitro. Immunostaining for the endothelial marker CD31 and Lectin demonstrated the growth of blood vessels in 10-week-old organoids cultured in vitro (Figure 5C). For the grafts transplanted in vivo for 12 months, we observed that the CD31-positive vessel-like structures were co-immunostained with *human* nuclei, indicating vascularization from the host environment after transplantation (Figure 5D,E). These results demonstrate the successful vascularization of cerebral organoids both in vivo and in vitro.

## 4. Discussion

In this study, we cultured and differentiated hiPSCs into cerebral organoids in vitro using a feeder-free culture system. Then, the obtained cerebral organoids were transplanted as small clusters, and their survival and maturation were evaluated at 12 months post-transplantation. We found that the transplants could survive well in the host brain for an extended period. Moreover, the grafted cerebral organoids maintained their forebrain identity, had a low proliferative capacity, and most of them matured into cortical neuronal subtypes in different cortical layers and GABAergic neurons. In addition, a small population of grafts differentiated into astrocytes, microglia and oligodendrocytes. Furthermore, synaptic formation and vascularization were observed between the grafted and host cells.

To date, the majority of xenotransplantation studies have used cell-dense transplants containing a large number of cells, which have limited access to the host tissue [27]. For cerebral organoid transplantation, to maintain structural integrity, most studies have performed transplantation surgery to create a cavity and injected several organoids directly into the cavity [15]. It is possible that these transplants may form a local microenvironment that is different from that of the host brain. In addition, these oversized organoids may compress the surrounding healthy brain tissue, which may reduce the therapeutic effect of the transplant. Linaro et al. [28] explored the xenotransplantation of *human* cortical neurons as single cells into the *mouse* cortex. They found that the transplanted single cells matured and became highly integrated with the host brain. In our study, we dissociated whole cerebral organoids into single cells and transplanted the resulting small clusters after one day of aggregation. We found that the transplanted cells could grow in a dispersed manner, survive, and mature well in vivo over a long period. In Revah’s study, the authors transplanted whole organoids into the *rat* cortex using a 23 G needle, and then assessed the cytoarchitecture and cellular composition by immunostaining for SATB2 and CTIP2 at 8 months post-transplantation. They found that, despite the presence of cortical layer subtypes, the transplanted cerebral organoids displayed no obvious anatomical lamination [29]. In our study, we found that the TBR1- and CTIP2-positive neurons were mainly distributed in the deep cortical layers, while the SATB2-positive neurons were mostly distributed in the upper cortical layers. We speculate that this anatomical lamination is mainly attributable to the migratory characteristics inherent in neuronal maturation. It is unclear whether this migration is associated with the transplantation of small clusters, which enabled cells to migrate more easily out of the grafts.

Previous studies have demonstrated that *human* neurons mature more slowly than *mouse* neurons in vivo [30,31]. The studies published so far have explored the survival and maturation of cerebral organoids over periods ranging from several days to several months. Kitahara et al. [13] showed that engrafted 10-week-old cerebral organoids expressed 60.6% SATB2-positive cells, 6.1% CTIP2-positive cells, and 2.7% Ki67-positive cells at 3 months post-transplantation. Moreover, they also demonstrated that the engrafted cerebral organoids extended a large number of axons along the host corticospinal tract and became vascularized within the host. One study analyzed the functional maturation of transplanted cerebral organoids using electrophysiology at 5 months post-transplantation. However, the cellular composition of the grafted cells was not mentioned at this stage [16]. In our study, we analyzed the cellular composition of grafted cerebral organoids at 12 months post-transplantation. The results indicated that most transplanted cells matured into cortical neurons across different layers. Fewer than 1% Ki67-positive cells in the engrafted cerebral organoids indicated a low risk of tumorigenesis.

In the mammalian cortex, GABAergic neurons form the major inhibitory system and are crucial in the organization and function of neural circuits [32,33]. GABA-positive cells can be obtained in forebrain organoids after day 84 in vitro [12]. In our culture system, GABA-positive cells were also obtained and distributed in different layers of the transplanted cerebral organoids. We found that 15.87% ± 2.49% of cells in the transplanted cerebral organoids were GABA-positive. We speculate that this population of GABAergic neurons may play an important role in shaping excitatory and inhibitory neuronal networks. However, we have not yet distinguished the subtypes of GABAergic neurons (e.g., PV^+^, SST^+^, VIP^+^), and their intrinsic electrophysiological properties and functions were not assessed. We expect to further explore these aspects in future research.

Glial cells, including astrocytes, microglia, and oligodendrocytes, play an important role in cerebral development. Astrocytes are the most abundant glial cells in the central nervous system and are involved in neurotrophic support, information processing, and neural circuit integration [34]. Oligodendrocytes can wrap glutamatergic projection neurons and GABAergic inhibitory neurons to form myelin sheaths during cortical development [35]. Myelination enables the saltatory conduction of action potentials. In addition, oligodendrocytes play an important role in sustaining and modulating neuronal function [36]. Microglial cells are the professional phagocytes of the mammalian brain and play the important role of eliminating entire cells or cellular components, which ensures the integrity of neuronal plasticity [37]. Therefore, the generation of glial cells, including astrocytes, microglia, and oligodendrocytes, in grafted cerebral organoids is fairly important for the establishment of neural circuits. Revah et al. [29] observed the expression of GFAP and IBA1 at 8 months post-transplantation of grafted cerebral organoids. However, they did not detect MBP expression in the transplanted organoids. In our study, we observed the expression of markers for astrocytes, microglia, and a very small number of oligodendrocytes both in vitro and in vivo. We suggest that these glial cells may promote the integrity of neuronal circuits in cerebral organoids.

The maturation of the grafted cells in vivo mainly involves both structural and functional aspects. Previous studies have examined the maturation and functional integration of transplanted *human* cortical organoids in vivo through immunostaining, electrophysiological recordings, and animal behavioral tests [16,29]. In these studies, the authors demonstrated that the grafts could mature into different cell subtypes, form synapses and achieve vascularization with host cells. Furthermore, they also indicated the functional integration of the grafts with host cells after 3–8 months post-transplantation. In our previous study, we demonstrated that synaptic connection between grafts and host cells occurred as early as 2 months post-transplantation, and these newly formed synaptic connections were sequentially but not synchronously functionally mature in vivo [38]. Therefore, we speculate that the long-term survival and maturation of grafts is essential for them to be able to perform therapeutic functions. In addition, vascularization is essential for the survival and functional exertion of grafts in vivo [13,19]. In our study, we further demonstrated that cerebral organoids can be vascularized both in vivo and in vitro. We consider that vascularization holds great importance for the clinical application of brain organoids as cell therapy resources. Previous studies have demonstrated that grafted organoids can mature at the electrophysiological level after several months post-transplantation. Unfortunately, in our current study, we did not conduct functional validation at the electrophysiological or behavioral levels. We hope that, in future research, we will be able to validate these results in animal models of diseases such as stroke, hypoxic–ischemic encephalopathy, and traumatic brain injury.

## 5. Conclusions

In conclusion, we demonstrated that it is feasible to dissociate cerebral organoids into single cells and transplant the aggregated small clusters into *rodent* brains. The transplanted small cerebral organoids survived well over a long period in the *mouse* cortex. The majority of the grafted cells matured into cortical neuronal subtypes across different layers and GABAergic neurons. In addition, a small population of grafted cells matured into glial cells, including astrocytes, microglia, and oligodendrocytes. Furthermore, synapses and vascularization were formed between the grafted and host cells. Our study illustrates the feasibility of stem cell transplantation using cerebral organoids derived from hiPSCs and lays a foundation for cerebral organoid-based therapeutic applications in neurological diseases.

## Data Availability

The original contributions presented in this study are included in the article/Supplementary Materials. Further inquiries can be directed to the corresponding author.

## Supplementary Materials

The following supporting information can be downloaded at: https://www.mdpi.com/article/10.3390/cells15161466/s1, Figure S1: Characterization of hiPSCs line. (A) Representative immunoflorescent images showing the expression of stem cell makers OCT4, NANOG, SSEA4 and SOX2. Figure S2: Temporal gene expression of cerebral organoids derived from hiPSCs at different stage: (A) Neural progenitor cells. (B) Cortical neuron subtypes. (C) Astrocytes and cortical glutamatergic neurons. D = day. Figure S3: Characterization of cerebral organoids derived from hiPSCs at day35 in vitro: (A) Representative immunofluorescent images showing the expression of SOX2, Ki67, PH3, PAX6 and TBR2 in cerebral organoids at day 35. (B,C) Representative immunofluorescent images showing the expression of MAP2 and CTIP2 in cerebral organoids at day 35. Figure S4: Distribution of transplanted cells within the host brain. (A–C) Representative immunofluorescent images showing the expression and distribution of CTIP2, TBR1 and SATB2 in transplanted cells. Table S1: Antibodies used for all immunofluorescence assays. Table S2: Primer sequences of RT-PCR.
