# Supplementary material for: Long-Term Survival and Maturation of Transplanted Cerebral Organoids Derived from Human Induced Pluripotent Stem Cells
Source: Cells. 2026 Aug 15;15(16):1466. doi: 10.3390/cells15161466 (PMC13511031; doi:10.3390/cells15161466)
Supplement: Supplementary file 1 [file cells-15-01466-s001.zip › cells-4431262-supplementary.pdf]

Supplementary Information

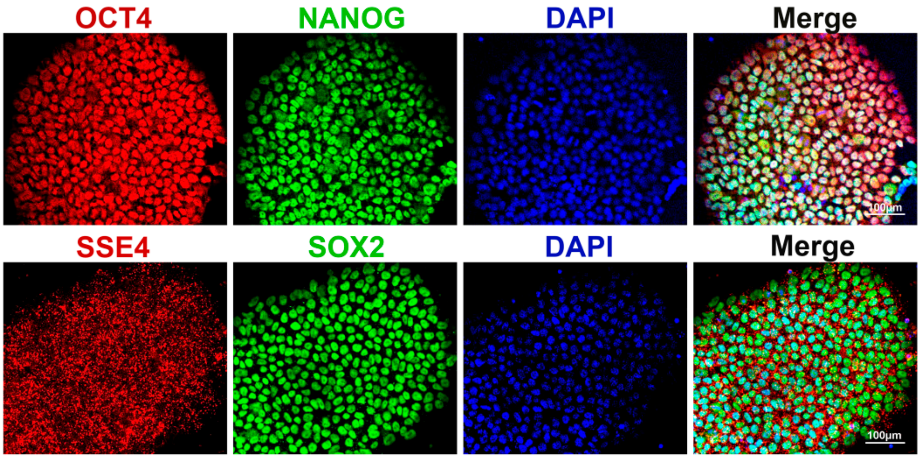

Figure S1: Characterization of hiPSCs line. (A) Representative immunoflorescent images showing the expression of stem cell makers OCT4, NANOG, SSEA4 and SOX2.

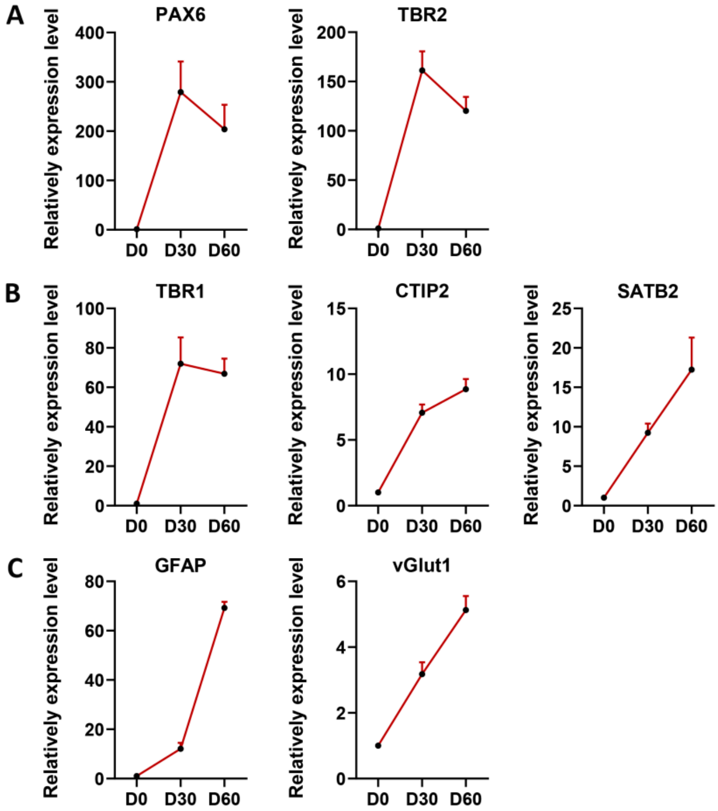

Figure S2: Temporal gene expression of cerebral organoids derived from hiPSCs at different stage: (A) Neural progenitor cells. (B) Cortical neuron subtypes. (C) Astrocytes and cortical glutamatergic neurons. D=Day.

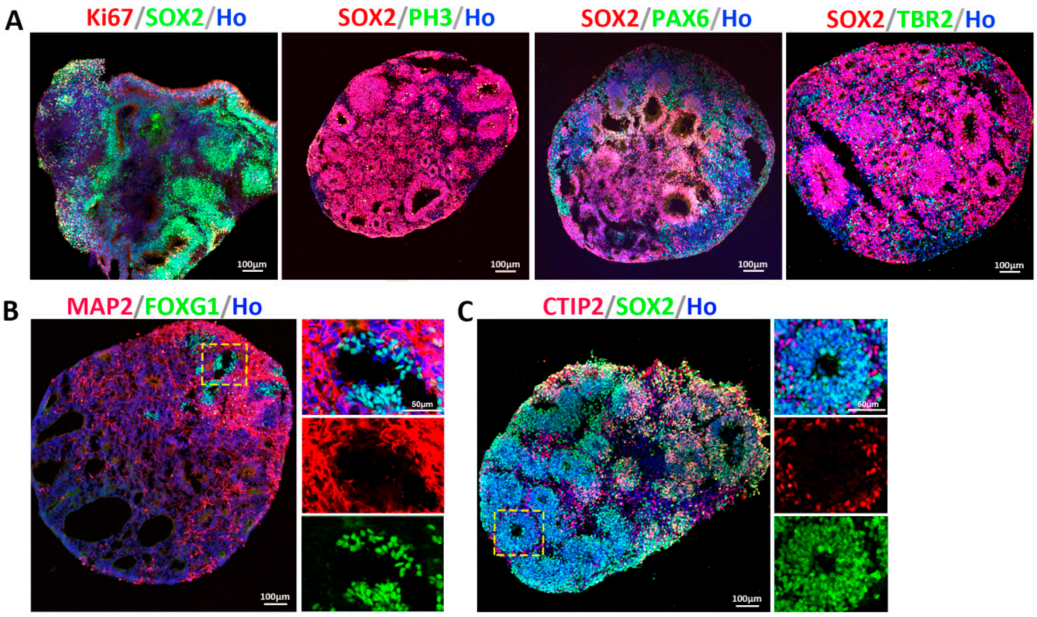

Figure S3: Characterization of cerebral organoids derived from hiPSCs at day35 in vitro: (A) Representative immunofluorescent images showing the expression of SOX2, Ki67, PH3, PAX6 and TBR2 in cerebral organoids at day 35. (B, C) Representative immunofluorescent images showing the expression of MAP2 and CTIP2 in cerebral organoids at day 35.

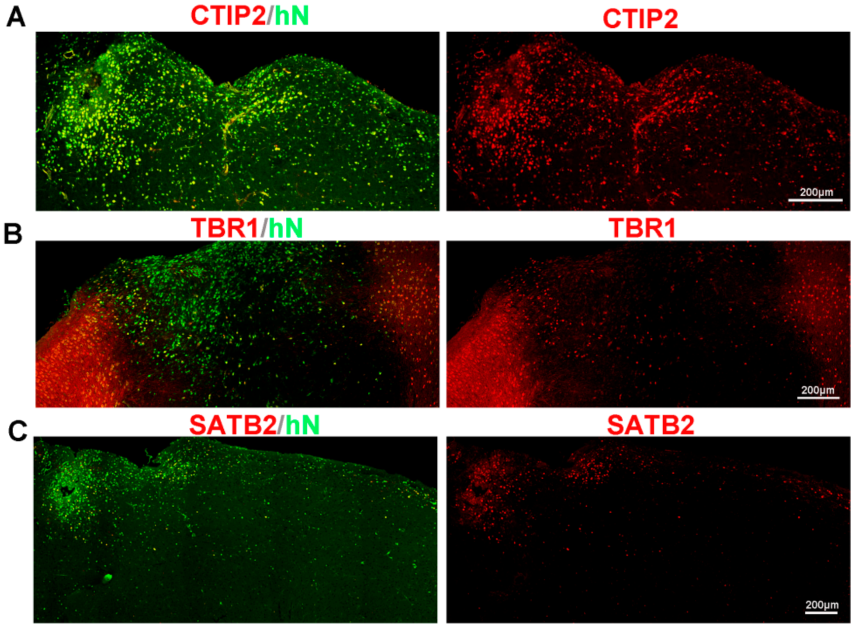

Figure S4: Distribution of transplanted cells within the host brain. (A-C) Representative immunofluorescent images showing the expression and distribution of CTIP2, TBR1 and SATB2 in transplanted cells.

Table S1. Antibodies used for all immunofluorescence assays

Type	Target	Cat.No	Manufacturer	Host	Conjugate	Dilution
Primary	FOXG1	ab18259	Abcam	Rabbit		1 in 800
Primary	PAX6	901301	Biolegend	Rabbit		1 in 800
Primary	SATB2	ab92446	Abcam	Rabbit		1 in 800
Primary	CTIP2	ab28448	Abcam	Rabbit		1 in 1000
Primary	TBR1	ab31940	Abcam	Rabbit		1 in 800
Primary	GABA	A2052	Sigma-Aldrich	Rabbit		1 in 4000
Primary	SOX2	AF2018	R&D Systems	Goat		1 in 500
Primary	Ki67	ab15580	Abcam	Rabbit		1 in 500
Primary	TUJ1	T2200	Sigma-Aldrich	Rabbit		1 in 2000
Primary	Synaptophysin	NBP1-19222	Novus	Mouse		1 in 1000
Primary	PSD95	48638	Signalway Antibody	Rabbit		1 in 200
Primary	GFAP	Z0334	DAKO	Rabbit		1 in 5000
Primary	Iba1	Ab178846	Abcam	Rabbit		1 in 1000
Primary	PKCλ	610207	BD	Mouse		1 in 300
Primary	human Nuclei	MAB1281	Millipore	Mouse		1 in 500
Primary	O4	MAB1326	R&D Systems	Mouse		1 in 500
Primary	CD31	62240	Signalway Antibody	Rabbit		1 in 500
Primary	VGLUT1	135303	Synaptic Systems	Rabbit		1 in 1000
Primary	HOPX	sc-398703	Santa Cruz	Mouse		1 in 500
Primary	CUX1	sc-514008	Santa Cruz	Mouse		1 in 500
Primary	BRN2	sc-514008	Santa Cruz	Mouse		1 in 100
Primary	MBP	ab7349	Abcam	Rat		1 in 300
Primary	MAP2	M1406	Sigma-Aldrich	Mouse		1 in 1000
Secondary	Mouse IgG	A21202	Invitrogen	Donkey	AlexaFluor 488	1 in 800
Secondary	Rabbit IgG	A10042	Invitrogen	Donkey	AlexaFluor 568	1 in 800
Secondary	Mouse IgG	A10037	Invitrogen	Donkey	AlexaFluor 568	1 in 800
Secondary	Rat IgG	A21209	Invitrogen	Donkey	AlexaFluor 594	1 in 800
Secondary	Goat IgG	A11057	Invitrogen	Donkey	AlexaFluor 568	1 in 800

Secondary	Rabbit IgG	A31573	Invitrogen	Donkey	AlexaFluor 647	1 in 800
Secondary	Mouse IgG	A31571	Invitrogen	Donkey	AlexaFluor 647	1 in 800
Secondary	Goat IgG	A21447	Invitrogen	Donkey	AlexaFluor 647	1 in 800
Secondary	Rat IgG	712-605-153	Jackson	Donkey	AlexaFluor 647	1 in 800
Lectin		DL-1174	Vector Laboratories		AlexaFluor 488	1 in 300
Hoechst 33342		14533	Sigma-Aldrich			1 in 2000

Table S2. Primer sequences of RT-PCR

Gene		Primer sequence
PAX6	Forward	TGGGCAGACAGCATGTATAAC
PAX6	Reverse	CTGGTAGGTACCAGAGGAAGTG
TBR2	Forward	GGCAGCTGGACTACCATGGA
TBR2	Reverse	TGAGATCTCCAACTTTCCTCTTGG
TBR1	Forward	CAAGTACCAGCCCCGCCTG
TBR1	Reverse	GCTGGTAGTCCTCCGTGCC
CTIP2	Forward	CAGAGCAGCAAGCTCACG
CTIP2	Reverse	GGTGCTGTAGACGCTGAAGG
SATB2	Forward	CACCAGCAGCCCTCATGAGT
SATB2	Reverse	GCCAGCGTTCCACACCATAC
GFAP	Forward	GAGAGGAGACGCATCACCTCC
GFAP	Reverse	CGGATGGTGATCCTTCTCGC
VGLUT1	Forward	GCGAGACCGTAGGAATACTCG
VGLUT1	Reverse	CAGGCCACCTTGCTGATCTC
GAPDH	Forward	AATGGGCAGCCGTTAGGAAA
GAPDH	Reverse	GCGCCCAATACGACCAAATC
